# Envisioning and shaping translation of knowledge into action: A comparative case-study of stakeholder engagement in the development of a European tobacco control tool

**DOI:** 10.1016/j.healthpol.2019.07.012

**Published:** 2019-10

**Authors:** Robert A.J. Borst, Maarten Olivier Kok, Alison J. O’Shea, Subhash Pokhrel, Teresa H. Jones, Annette Boaz

**Affiliations:** a Erasmus School of Health Policy & Management, Erasmus University Rotterdam, P.O. Box 1738, 3000 DR, Rotterdam, the Netherlands; b Amsterdam Public Health research institute, Department of Health Sciences, Faculty of Science, Vrije Universiteit Amsterdam, De Boelelaan 1085, 1081 HV, Amsterdam, the Netherlands; c Faculty of Health, Social Care and Education, A partnership between Kingston, University London and St George’s University of London, Cranmer Terrace, London, SW17 0RE, United Kingdom; d Health Economics Research Group, Institute of Environment, Health and Societies, Brunel University London, Kingston Lane, Uxbridge, Middlesex, UB8 3PH, United Kingdom

**Keywords:** Stakeholder engagement, Actor-scenario mapping, Research impact, Context, Knowledge translation

## Abstract

Stakeholder engagement in health policy research is often said to increase ‘research impact’, but the active role of stakeholders in creating impact remains underexplored. We explored how stakeholders shaped the translation of health policy research into action. Our comparative case-study tracked a European research project that aimed to transfer an existing tobacco control return on investment tool. That project also aimed to increase its impact by engaging with stakeholders in further developing the tool. We conducted semi-structured interviews, using an actor-scenario mapping approach. Actor-scenarios can be seen as relational descriptions of a future world. We mapped the scenarios by asking stakeholders to describe who and what would play a role in the tool’s utilisation. Our results show that stakeholders envisioned disparate futures for the tool. Some scenarios were specific, whereas most were generic projections of abstract potential users and responsibilities. We show how stakeholders mobilised elements of context, such as legislative support and agricultural practice, that would affect the tool’s use. We conclude that stakeholders shape knowledge translation processes by continuously putting forth explicit or implicit scenarios about the future. Mapping actor-scenarios may help in aligning knowledge production with utilisation. Insights into potential roles and responsibilities could be fed back in research projects with the aim of increasing the likelihood that the study results may be used.

## 1 Introduction

The practice of stakeholder engagement in knowledge production is gaining increasing traction in research funding debates [[1], [2], [3]]. One of the principal reasons for engaging stakeholders is that it might increase the likelihood that research outputs will be used [4,5]. In practice, researchers often retrospectively attribute the use of their findings to their engagement with stakeholders [6]. Others emphasise the importance of prospectively exploring how stakeholder engagement processes evolve and affect the translation of knowledge into action [7]. How stakeholder engagement shapes the use of knowledge, and which roles stakeholders play in this, had been largely underexplored [8,9].

The literature suggest that stakeholder engagement affects knowledge translation in different ways. First, stakeholders may add valuable knowledge and skills to the research process [10,11]. Second, stakeholders possess experiential information about the environment in which the research findings might be used. Such information can be used to align the research process with the environment in which the research findings could be used [7]. Third, by being engaged, stakeholders gain a better understanding of the prospective study results. This would inform the stakeholders of the study taking place, but also encourages them to think about potential use of the results in practice [12,13]. Finally, engagement can establish a trust-relationship between researchers and potential users. Trust is essential for mutual understanding and communication between actors and increases the presumed legitimacy of results [14,15]. Oliver, Kothari and Mays [16] conclude that stakeholder engagement is generally considered to make a positive contribution to research projects, but may induce challenges and costs as well. In particular, they call for more reflection on when to engage stakeholders in research and in what way [16].

Science and technology studies (STS) emerged as a constructivist interdisciplinary field in the late 1970s and is known for studying knowledge production practices and the role that users play in these processes [[17], [18], [19]]. It seems particularly well equipped to reflect on the role of stakeholder engagement in health policy research. STS scholars offer a conceptualisation of ‘translation’ that is different to those commonly used in health policy literature [20,21]. Much of this conceptualisation is grounded in what Callon [22] calls ‘sociology of translation’ and which later became known as actor-network theory [23]. According to this conceptualisation, knowledge translation can be seen as a process of (political) activities by which actors actively displace and transform knowledge [24]. From such an understanding, translation is about negotiation, transformation, and the associations between actors through which networks are built and extended [23]. The strength of this understanding is that it offers an in-depth understanding of the active role of potential knowledge users in translation, the work that is necessary to make knowledge usable, the role of non-human actors (e.g. material environments), and a specific conceptualisation of the role of context in translating knowledge into action [17,25].

A theoretical aspect that remains underexplored in the literature on stakeholder engagement is how stakeholders themselves envision translation of knowledge into action [26]. In particular a focus on potential users and the role they play in shaping knowledge use could increase understanding of how stakeholder engagement affects knowledge translation processes. Stakeholders’ perspectives on translation of study findings into action can offer insight into the world in which the findings might be used, including necessary roles and responsibilities. Stakeholders can bring forward different accounts of the future world, with different roles and responsibilities [27].

To scrutinise how stakeholder engagement in knowledge production shapes the use of such knowledge in practice, this study prospectively followed stakeholder engagement in a large research project funded by the European Commission (EC). The European-study on Quantifying Utility of Investment in Protection from Tobacco (henceforth: project) centred around the transfer of an evidence-based tobacco return-on-investment (ROI) tool (see Box 1). The project explicitly planned to engage with stakeholders to increase the project’s ‘impact’ [28]. The case-study at hand was part of the parallel Stakeholder Engagement in EQUIPT for Impact (SEE-Impact) study. Our aim was to envision how stakeholder engagement shapes the translation of the ROI tool into action by mapping how stakeholders themselves put forward scenarios about the potential use of the ROI tool. It is anticipated that the findings of this study will contribute to the development of stakeholder engagement in research as a method for supporting research use.

Box 1 **Description of the SEE-Impact study in relation to the EC-funded EQUIPT project**

**Studying engagement in the development of a tobacco-control tool**

The project under study was funded through the European Commission’s Seventh Framework Programme. The European-study on Quantifying Utility of Investment in Protection from Tobacco (EQUIPT) was a collaboration between 11 members from seven countries (i.e. Belgium, Croatia, Germany, Hungary, the Netherlands, Spain, and the UK), and was led by the Health Economics Research Group (HERG) from Brunel University London. The project commenced in October 2013 and ended September 2016. Their aim was to assess the “cross-context transferability of economic evidence on tobacco control” which led them to further develop an existing ROI tool for use in other EU countries. As part of their project, they tested the tool in the Netherlands, Germany, Spain, and Hungary [28].

The existing tool had been developed in the UK by the HERG in conjunction with the National Institute for Health and Care Excellence (NICE) and is available on the NICE website (http://bit.ly/tobacco-roi). The tool allows users to calculate savings for every monetary unit invested in certain tobacco-control or smoking cessation interventions. The stakeholder engagement in the EQUIPT project was informed by the successful stakeholder engagement in the original UK project. This had contributed to the original ROI tool becoming the NICE’s support tool for English local authorities, which eventually informed the smoking cessation approaches of several local authorities.

The qualitative case-study presented in this paper was part of the Stakeholder Engagement in EQUIPT for Impact (SEE-Impact) study, funded by the Medical Research Council in the United Kingdom. SEE-Impact prospectively tracked all stakeholder engagement activities in EQUIPT with the aim of describing to what extent engagement affects research impact. The SEE-Impact study collected data through literature review, surveys, semi-structured interviews, and observations.

Alt-text: Box 1

## 2 Methods

For this in-depth case-study, we drew on data from 21 ethnographic interviews in Hungary and the Netherlands that were conducted as part of the SEE-Impact study. These two countries were part of the four countries (i.e. Hungary, the Netherlands, Spain, and Germany) to which the European research project aimed to transfer their tobacco ROI tool. For the purpose of our study, we selected Hungary and the Netherlands as contrasting cases [29]. These countries have very diverse socioeconomic and political contexts relevant to health policy. Particularly relevant to this study is the countries’ difference in tobacco policies and smoking prevalence [30,31]. At the time of this study, Hungary had more stringent tobacco control policies than the Netherlands, but a higher smoking prevalence [32,33]. More information on the SEE-Impact study and its methods can be found elsewhere [9].

### 2.1 Interviewees

We sampled stakeholders with different levels of engagement. The first group of stakeholders concerned partners of the EQUIPT project. The second group consisted of actors that were invited by the EQUIPT project to provide input in the continued development of the tool. The final group included actors who could have been approached by the EQUIPT researchers (i.e. they belonged to similar networks as the second group), but with whom no interaction had occurred. We selected the final group of actors based on their substantive experience in tobacco control or health policy within each country.

### 2.2 Data collection and analysis

A total of 21 interviews with eight Hungarian and eleven Dutch stakeholders were conducted. The interviewees were mostly academics working in health policy, health technology assessment, or epidemiology (n = 10), followed by government officials and parliamentarians (n = 5), and clinicians (n = 4).

We used a theoretical framework (see Box 2) to guide the ethnographic interviews. In particular, we developed topic lists that specifically sought to map actor-scenarios by asking interviewees to think of how the tool would be used in the future and who would play a role in that use. This approach added some structure to the interviews, but allowed for a subjective, anticipatory, exploration of topics that did not directly align with the concept of actor-scenarios [37]. During data collection, three topics that were regularly mentioned in the scenarios were added to the lists (i.e. decentralisation of public services, earlier experience with stakeholder engagement, and politics). All interviews were audio recorded and the interviewers kept detailed notes during the interviews. Immediately after each interview, reflectional memos were prepared and recordings were transcribed verbatim.

Box 2 **Description of actor-scenario mapping as an approach to studying the use of knowledge**

**Actor-scenario mapping**

Building on Michel Callon’s notion of ‘actor worlds’ [24], we use the concept ‘actor-scenario’ to refer to the process of actors implicitly or explicitly putting forth scenarios about practices in a future world [27,34]. An actor-scenario can be seen as a relational description of potential practices, roles, and responsibilities. Actor-scenarios are fictive at first, but performative as well since they include descriptions of what should happen for the scenarios to be enacted [35,36]. The practice of scenario-building works as ongoing negotiation process through which actors aim to effectuate change [24,37]. Researchers, for instance, constantly put forth implicit or explicit accounts of the role that their findings should play in a future world.

Different actors may construct different scenarios that each portray their own roles and responsibilities. Some parts of the scenarios might overlap, whereas others diverge. The actors that are enrolled in the scenarios can also refute their role and produce a different scenario with other roles and responsibilities. One of such roles might be reserved for knowledge, for instance to strengthen a scenario or weaken scenarios of others [34]. Mapping the actor-scenarios of the stakeholders in the EC-funded project may explicate who the stakeholders think should use the tool, how the tool should be used, and under which circumstances use is possible. We developed ‘actor-scenario mapping’ as an approach to envision and describe possible translations of the ROI tool into action.

Alt-text: Box 2

The process of data collection and analysis was conducted iteratively. This approach allowed the researchers to identify emerging themes suitable for subsequent fieldwork. Actor-scenario mapping uses an abductive sequence of analysis that requires constant shifting between theory and empirical findings [38]. The aim is to synthesise the different scenarios and offer thick descriptions of potential translations, including the different envisioned roles and responsibilities. The potential translations in this study were arrived at through repeated in-depth coding sessions with all team members.

### 2.3 Research ethics

The data collection of this study adhered to the Declaration of Helsinki and ethical clearance was obtained from Kingston University London’s Faculty Research Ethics Committee (FREC 2014/01/011). Accordingly, the researchers obtained written informed consent of the interviewees and the interviewers explicitly stated that the anonymised results would be published.

### 2.4 Study schedule

This study was conducted between February 2015 and March 2017. The data collection was carried out between April 2015 and September 2016.

## 3 How stakeholders envisioned the tool to be used

The envisioned uses of the tool were situated and shaped by local-specific dynamics and elements of context. Conventional with actor-network theory, we will provide separate descriptive accounts of how Hungarian and Dutch interviewees envisioned the use of the tool. We will start each section with describing the roles and responsibilities put forth by the stakeholders, followed by what the stakeholders described as potential enabling or constraining elements of context in the use of the tool.

### 3.1 The potential users in Hungary

The actor-scenarios of Hungarian stakeholders were often quite similar. The Hungarian stakeholders, for example, all designated the National Focal Point for Tobacco Control a role as user. The focal point, they described, would be a suitable user because of their experience with economic evaluations and embeddedness within the official health system. Several of the interviewees spoke of a specific person within the focal point. They described how this person could use the tool to compare interventions on their cost-effectiveness, and how *“he feeds the Ministry with his data.” (Clinician 1)*. They also stressed the importance of the focal point being appointed by the government. This – combined with the focal point’s status as WHO partner office – would legitimise their recommendations amongst policymakers.

Some stakeholders assigned the National Institute of TB and Pulmonology a role in their scenarios. An interviewee working at the National Public Health and Medical Officer’s Service (ÁNTSZ) described that they did a lot of their smoking cessation activities together with the National Institute. The interviewee described that the National Institute is very active in this field and would likely be interested in the tool. When we asked one of the Institute’s employees whether they would use it, the interviewee said that they *“would tell about it [the tool] and (…) would teach with it.” (Clinician 1)*

In addition, stakeholders commonly mentioned the National Health Insurance Fund. While nearly all respondents assigned the Fund some role in their scenarios, they articulated different responsibilities. An epidemiologist spoke of the Fund as the *“most likely user”* and described that the Fund could use the tool’s output as *“ammunition to argue for some services to be reimbursed”.* A clinical professor argued that the Fund may use an efficacy comparison of smoking cessation programmes, although this would still be *“a bit further away from their focus”.* Others explained that the Fund could provide financial data but would otherwise not be interested in tobacco issues. The scenario of a former Fund employee resembled scepticism about the Fund’s responsibilities:

*“They would, directly, not be interested; even if officially they need to be interested. (…) Frankly, they are going to have a new intervention that would need to be reimbursed. So, their budget will be lower. (…) In case it is cost-saving; then it is fine. But, that will probably not happen. It will not be cost-saving.”* (Academic 1)

Few interviewees mentioned the Secretariat of Health as a possible user of the tool. Those who did, described that policymakers inside the Secretariat could use the tool to prioritise their decisions on which interventions to implement.

### 3.2 Envisioned translation in Hungary

Throughout the interviews in Hungary, a pattern emerged showing how elements of context would enable or constrain the potential use of the tool. Most interviewees articulated identical elements of context, commonly referring to the newly enacted tobacco legislation of 2012. A respondent that was involved in writing the 2012 legislation, explained the strategic work necessary to establish it:

*“We calculated: it was December, the first time that we could reach the Parliament would be mid-April. We did not trust our system–in a way that this voice went out early on last time. So, we did the professional work–the planning of the law–and then the State Secretary discussed it inside the Parliament. What happened was that the law, planned and written, was given to Parliament where a group of parliamentarians said together:‘we are from the leading party and we think it is a very important public health problem in Hungary, we must change it, now!’And in two weeks’time, it was voted on. That was probably the only law, in the light of years, where left wing and right wing, whatever wing, they all voted. And it was something close to a ninety percent positive vote.”* (Clinician 1)

Several respondents explained that with the legislation’s enactment, tobacco retail was restricted, smoking in confined spaces prohibited, and excise taxes were increased. They stated that there is no need for a ROI tool, as there is barely room left for additional interventions.

*“If you evaluate the actual situation in Hungary, we achieved practically everything. There is no space. So, we are at the top, if related to legislation. But, there were some concerns that your private car is a confined space.”* (Clinician 2)

Another dynamic that some of the interviewees mentioned was the decentralised and segmented government. The respondents illustrated that there is a tension between two organisations both operating at the local level. On the one hand, there are the county public health departments, run by the county government offices and directed by the Prime Minister’s Office; on the other hand, there are the municipal health promotion offices administered by the ÁNTSZ on behalf of the Secretary of Health. One of the respondents explained that these organisations’ similar responsibilities cause regular tensions.

“*They are separated and there are conflicts. Because, they are working on similar issues. The conflicts are because they don’t really like each other to work on the same issue*.” (Government official 1)

Additionally, several participants described that the tool’s use might be constrained by the prominent place tobacco agriculture takes in Hungary. They described that the Ministry of Agriculture has a prevailing role in Hungarian policymaking. Besides, this Ministry’s main interest would be the tobacco cultivation in the North-Eastern part of Hungary.

“*The Ministry of Agriculture, for example, is very much opposed to regulating tobacco. Because they think that, I do not know, these few thousand people should grow tobacco and nothing else. I never understood why not to grow paprika instead, but okay*.” (Academic 2)

Another respondent explained that it is a concurrence of several circumstances that complicates the translation of evidence into anti-tobacco policies. The interviewee described how actors such as the educational system, soil, precipitation, temperature, and money position themselves as *“tobacco allies”* and constrain the enactment of anti-tobacco policies.

“*Tobacco policy depends practically on the agricultural tradition of the country. So, you need a special soil to grow tobacco, and the special circumstances related to temperature, precipitation, and so on. The best region for tobacco plantations in this country is the least-developed part, namely: The North-Eastern part.*” (Clinician 2)

An element that appeared to be linked to the tobacco agriculture was Hungary’s history of communism. A former politician explained that excessive smoking was a common habit during the Soviet era. During service in the Red Army, the respondent would receive a daily amount of 15 cigarettes regardless of whether one smoked or not. These cigarettes would be supplied by State-run tobacco plantations as part of the planned economy. After the fall of communism, the proprietorship was transferred to the corporate tobacco industry. The interviewee said that it was only by then that the medical community first initiated an anti-tobacco community with the philosophy to reduce tobacco-related harm.

Stakeholders in Hungary regularly spoke of the same actors in their scenarios about the potential use of the tool. Some stakeholders constructed slightly different scenarios. Overall, there seemed to arise convergence in stakeholders’ narratives about enabling and constraining dynamics in the potential use of the tool.

### 3.3 The potential users in the Netherlands

Unlike in Hungary, the actor-scenarios of Dutch stakeholders showed divergence. Interviewees described that the tool would not be used at all, or that its use would be constrained by what was referred to as ‘the political climate’. Sometimes participants mentioned specific organisations, but usually expressed uncertainty as to whether these organisations would actually use the tool. All interviewees assigned ‘policymakers’ a role in their scenarios, but without specifying who this actor is in practice. While some scenarios were more specific, most stakeholders did not articulate what the role and responsibility of policymakers specifically would be.

Interviewees commonly said that the municipal government and Municipality Health Service (GGD) would play a role. One professor in health policy explained that the municipal government might use the tool to guide their service procurement. Two tobacco control experts, however, described that anti-tobacco incentives are not the municipality’s priority; their political accountability causes them to prioritise less sensitive issues. The experts described that the municipal governments do not allocate the GGDs any anti-tobacco related tasks and that the GGDs do not have the resources to carry out anti-tobacco activities themselves. Even if they had, they would not have the expertise to use ROI tools for it, as one governance scholar explained.

Several respondents mentioned the National Institute for Public Health and Environment (RIVM) as a potential user. The RIVM functions as advisory body to the government. One interviewee explained that the RIVM as potential administrator could store the tool and update it if necessary. Two interviewees clarified that the RIVM used to deploy similar tools to answer tobacco control questions raised by the Ministry of Health. An interviewee formerly active in tobacco control shared an article that showed how the RIVM used to produce ‘scenarios’: predictions of the impact that certain combinations of anti-tobacco interventions could have.

*“It was not a tool in which everyone could twist the knobs, it was quite complex. (…) So, all these scenarios were already there. (…) Because, back then, the Ministry still gave the RIVM such orders.”* (Academic 3)

An interviewee working at the RIVM claimed to recognise that they no longer receive any orders from the Ministry to estimate the return-on-investment of tobacco control interventions.

*“The assumption of this European tool is that policymaking is mainly motivated by rational considerations; whereas in practice, that is obviously not the case. Such a tool could help to stimulate this, that makes sense. But, I am not sure whether the RIVM would use it to answer questions of the Ministry. That would mean that there is a situation in which a policymaker, at local or national level, says:‘we want to discourage the use of tobacco, this is the amount of funding, these are the conditions, now what would be the most efficient use of our resources?’Well, that is a laboratory situation that will never happen in practice.”* (Government official 2)

The interviewees disagreed on the role that researchers could play. One academic said that academic researchers would use the tool to evaluate the cost-effectiveness of interventions. Some of the project members planned for academics to adapt and update the tool. Two public health academics spoke of researchers at a national institute for mental health and addiction using the tool for monitoring. Nearly all interviewees, however, described that this activity would be a bit further from the institute’s core focus – since tobacco control is not included in their mandate.

Respondents often articulated generic ideas of who might be interested or capable in using the tool. Occasionally, these ideas were refuted by other respondents. Overall, there appeared to be multiple deviating scenarios about the potential use of the tool. The likelihood of the scenarios to be translated into action seemed to be affected by dynamics in the countries’ context. The elements that were mentioned by the respondents are portrayed in the subsequent sections.

### 3.4 Envisioned translation in the Netherlands

An element that prevailed in the scenarios of Dutch actors was the political climate. The majority of Dutch interviewees referred to two acts of the then Minister of Health in 2010. The first being her emphasis on ‘de-patronisation’ with regard to anti-tobacco legislation. The second act was the Minister’s repeal of the smoking ban for small restaurants and bars. One interviewee clearly remembered the Minister’s position on tobacco control:

*“We went to the Ministry in 2010 and offered the Minister a petition against tobacco, with over 1000 signatures. So, we visited her and she said:‘well, I really think this [smoking] is a free choice and I am not so fond of statistics.’That is what she said in that conversation. [raising voice]”* (Clinician 3)

Interviewees often spoke of the closing of the national expertise centre on tobacco control in 2013. A former employee explained that the Ministry of Health suspended its funding by 2011. Subsequently, the health foundations, responsible for the other half of the funding, decided to independently profile themselves more actively on tobacco control. The ex-employee explained that some of the activities were transferred to other organisations. Nonetheless, the majority of the centre’s promotional activities were abandoned and it remains unclear who should fill that gap.

While the respondents tried to identify potential users of the tool, they said that it is actually quite unclear who governs tobacco control in the Netherlands. One local government official explained that the Ministry of Health stipulates quadrennial national prevention priorities that should guide the municipal governments in prioritising at the municipal level. The municipality would then be officially responsible for the execution of prevention. But, several interviewees expressed that tobacco prevention might not be the municipalities’ uppermost priority.

When we asked whether politics would play a role, nearly all actors referred to political incentives. A Member of Parliament for the Labour party described that tensions within the then minority cabinet would prevent future anti-tobacco interventions from gaining traction. According to the interviewee, anti-tobacco policies do resonate within the Labour party, but the coalition agreement refrains them from acting. Other interviewees – who used to work on tobacco control for a longer period – seemed sceptical: they indicated that Parliamentarians would focus on increasing the government budget within the four-year cycle, and tobacco-control does not fit that agenda.

*“The current political landscape is fragmented and there is no majority for a more stringent policy on smoking. (…) There are actually two opposed sides: the conservative-liberalist side on which it is a freedom of choice, and the socio-democratic that says: tobacco is a perverse incentive of the government to complement the treasure chest.”* (Parliamentarian)

Finally, the respondents often spoke of a recent history full with major health system reforms. An interviewee believed that these left little room for further tobacco control legislation. The interviewee explained that the former Minister of Health implemented the ban on smoking in confined spaces and major reforms of the public health law. The interviewee’s predecessor completely reformed the healthcare system, whereas currently the focus is on redistributing power between health insurers and medical professionals. Anti-smoking did not have place on the political agenda.

## 4 Discussion

This study aimed to explore how stakeholder engagement in knowledge production shapes the use of such knowledge in practice. In order to explore this, we studied engagement of stakeholders in the continued development of a tobacco control return-on-investment tool. We asked the stakeholders to put forth an explicit scenario about which actors would use the tool and under which circumstances the tool could be used. Most stakeholders described that they found it difficult to identify potential users of the tool. While most stakeholders envisioned quite a local-specific scene, they set the stage with exceedingly generic potential users and responsibilities. An example was that most Dutch interviewees spoke of ‘policymakers’ as potential users of the tool but were mostly unable to identify these actors in practice.

Our mapping of actor-scenarios offers three observations relevant to stakeholder engagement in knowledge production. First, we have introduced a specific understanding of stakeholders’ role in knowledge production. We showed how stakeholders have implicit or explicit understandings of how, by whom, and under which circumstances, the produced knowledge may be used, or what makes the produced knowledge relevant and usable. By engaging the stakeholders, these renderings of a future world will start interacting with the scenarios of the knowledge producers, who themselves inscribe their produced knowledge with assumptions about the circumstances under which the produced knowledge may be used [34,39]. It is these interactions that will shape to some extent how the knowledge may be translated into action. This can be especially challenging when the actor-scenarios of stakeholders seem to diverge, as was the case in the Netherlands. The diverging actor-scenarios may induce disputes over how these diverse inputs of stakeholders will be treated in the production of knowledge or who will be ‘the user’ of the produced knowledge [14,19].

A second observation is that actor-scenario mapping can provide an empirical understanding of what knowledge use entails in practice. In particular, our approach reiterates that knowledge in itself does not have a univocal value, but requires active work in order to become ‘usable’. This work involves a clear articulation of what roles and responsibilities the knowledge requires. In the Netherlands for example, the stakeholders of the European project tried to identify an actor that would govern tobacco control. According to the interviewees, such an actor would be a prerequisite in the use of the tool. They explained that without this governing actor, it would be impossible to act upon the knowledge. Similarly, the Hungarian respondents constituted the focal point for tobacco control by describing how it would have to become the key user of the tool.

A final observation to consider is how actor-scenario mapping contributes to our understanding of the role of context in knowledge translation. Our findings show how actors constructed context by bringing forth a scenario of roles and responsibilities. This implies that context is not something clearly defined and fixed, but instead refers to a fluid and contingent network of elements [40]. In their scenarios, the stakeholders described that some actors and dynamics would constitute ‘contexts’ that could enable or constrain the use of the tool [41]. Dutch interviewees for instance spoke of the then Minister of Health, local governments, and historical legislative reforms. Stakeholders in Hungary would refer to the soil, precipitation, and a history of communist-rule that all constrain tobacco control. In their scenarios, the stakeholders constructed a fluid boundary between content and context to account for a difference between use and that what shapes potential use, and subsequently mobilised the actors they designated as context [17,42].

Several questions about stakeholder engagement in knowledge production remain. A first question is what the convergence or divergence of different actor-scenarios means. In Hungary, the scenarios appeared to be more specific and converging than in the Netherlands. The data suggest that some actors are embedded and entangled in networks in different ways; potentially relating more to local-specific actors [24]. Conversely, an understanding of divergence might be found in the generic user descriptions (e.g. policymaker). Shove & Rip suggest that the use of such universal labels results in a mismatch with the roles that actors construct in practice [43]. Although the stakeholders might all consider the policymaker to be a primary user, their identifications of this actor in practice are likely to differ. This contradiction creates a problem of translation when this generic notion of users is inscribed in the produced knowledge [19]. It has been described before how these inscriptions have performative effects: the inscription could for instance impair use by anyone other than this generic user [35,44]. Lastly, it is important to note here that in the UK, where the development of the ROI tool first originated, engagement with stakeholders – being actors with an explicit stake in both the development and use of the tool – had been a key element of the success of the project. This observation has implications for the nature of stakeholder engagement in research – in particular in so far stakeholder engagement is concerned as means to increase research use [45].

In this study we developed actor-scenario mapping as approach to studying knowledge translation practices. The notion of actor-scenarios has been used before and is embedded in a wider literature on scenario-building, fictive scripting, and the construction of actor-worlds [24,27,37,46]. We further developed the notion of actor-scenarios into an approach to envision knowledge translation practices more generally. What distinguishes actor-scenario mapping from other analytical approaches, including thematic analysis and a constant comparative method, is its emphasis on abduction, sensitivity to the sociomaterial environments, and focus on mobilisation of contextual elements [27,38,47,48]. Additionally, the approach recognises the situated nature of both the actor that puts forth the scenarios, and the scenarios themselves. An element of actor-scenario mapping that needs to be developed further is its capacity to guide translation of knowledge into action. In other sectors, scenarios of the future are often used to plan or anticipate these possible futures. While we recognise the inherent contingencies in knowledge translation, it may be possible to productively use actor-scenario mapping in existing stakeholder mapping exercises. The scenarios could be used as input to research projects. In the example of the European research project, the scenarios may have been used to align the knowledge production process (i.e. continued development of the tool) with the stakeholders’ envisioned utilisations of that knowledge.

## 5 Conclusion

Our analysis suggests that engaging stakeholders in knowledge production shapes the translation of that knowledge into action in different ways. Stakeholders constantly put forth implicit or explicit scenarios about which actors might use the knowledge, in which way, and under what circumstances. These actor-scenarios are fictive at first but have a performative working as well: through their engagement, the stakeholders’ scenarios contribute to how the knowledge is constructed and thus also what its use entails. Actor-scenario mapping may help in actively aligning research processes with the translations that stakeholders envision. The assumptions and expectations of roles, responsibilities, and potential use, explicated by mapping the actor-scenarios, could be fed back in the research project and might help in increasing the likelihood that results will be used. Additionally, our data contribute to a deeper understanding of the ‘context of use’ by showing how actors mobilise elements of context in their scenarios, and how such elements could enable and constrain the use of knowledge.

## Declaration of Competing Interest

None.
